# Quantum Dots Encapsulated with Canine Parvovirus-Like Particles Improving the Cellular Targeted Labeling

**DOI:** 10.1371/journal.pone.0138883

**Published:** 2015-09-23

**Authors:** Dan Yan, Bin Wang, Shiqi Sun, Xia Feng, Ye Jin, Xueping Yao, Suizhong Cao, Huichen Guo

**Affiliations:** 1 State Key Laboratory of Veterinary Etiological Biology and National Foot and Mouth Disease Reference Laboratory, Lanzhou Veterinary Research Institute, Chinese Academy of Agricultural Sciences, Xujiaping 1, Lanzhou, Gansu 730046, China; 2 College of Veterinary Medicine, Sichuan Agricultural University, Chengdu, Sichuan, 611130, China; Wuhan Bioengineering Institute, CHINA

## Abstract

Quantum dots (QDs) have a promising prospect in live-cell imaging and sensing because of unique fluorescence features. QDs aroused significant interest in the bio-imaging field through integrating the fluorescence properties of QDs and the delivery function of biomaterial. The natural tropism of Canine Parvovirus (CPV) to the transferrin receptor can target specific cells to increase the targeting ability of QDs in cell imaging. CPV virus-like particles (VLPs) from the expression of the CPV-VP2 capsid protein in a prokaryotic expression system were examined to encapsulate the QDs and deliver to cells with an expressed transferrin receptor. CPV-VLPs were used to encapsulate QDs that were modified using 3-mercaptopropionic acid. Gel electrophoresis, fluorescence spectrum, particle size, and transmission electron microscopy verified the conformation of a complex, in which QDs were encapsulated in CPV-VLPs (CPV-VLPs-QDs). When incubated with different cell lines, CPV-VLPs-QDs significantly reduced the cytotoxicity of QDs and selectively labeled the cells with high-level transferrin receptors. Cell-targeted labeling was achieved by utilizing the specific binding between the CPV capsid protein VP2 of VLPs and cellular receptors. CPV-VLPs-QDs, which can mimic the native CPV infection, can recognize and attach to the transferrin receptors on cellular membrane. Therefore, CPV-VLPs can be used as carriers to facilitate the targeted delivery of encapsulated nanomaterials into cells via receptor-mediated pathways. This study confirmed that CPV-VLPs can significantly promote the biocompatibility of nanomaterials and could expand the application of CPV-VLPs in biological medicine.

## Introduction

Bio-imaging is an indispensable technology in life science [1]. Fluorescence indicators with excellent physicochemical properties, such as remarkable brightness and photostability, further expand the scope of biological applications [2]. Inorganic fluorescent nanomaterials, such as quantum dots (QDs), have unique advantages over organic fluorophores, including long-term visualization, size-tunable light emission, improved signal brightness, resistance against photobleaching, and simultaneous excitation of multiple fluorescence colors [3–5]. In general, QDs are nanoparticles with diameters of 1–100 nm that are synthesized using inorganic elements, such as Cd, Se, Te, P, Zn, and S. The core of QDs is usually composed of CdSe, CdTe, InP, or InAs; and the shell is ZnS [6]. The optical characteristics of a QDs in biomarker imaging are as follows: wide absorption band; narrow emission band; different diameters have different emission wavelengths and do not overlap; good photostability; and the fluorescent intensity is higher than that of organic dye under the same excitation light intensity [7–10]. However, QDs have poor biocompatibility and low water solubility; in addition, toxic cadmium ions could be released and degraded in vivo [11–13]. These disadvantages restrict the application of QDs in living organisms.

Many recent progresses have been conducted to enhance the stability and biocompatibility of QDs [14]. The most common methods are surface functionalization strategies through the linkage of hydrophilic molecules on the surface of QDs or encapsulation of QDs in water-soluble materials [15–17]. Commonly used hydrophilic molecules include amphiphilic polymers, amphiphilic lipid molecules, and hydrophilic groups (e.g., alcoholic, sulfhydryl, and carboxyl groups) [18–21]. Water solubility and stability of QDs are increased, and bio-toxicity is reduced through the covalent modification of QDs surfaces [22]. However, temperature, pH value, concentration, and reaction time can greatly affect the repeatability of QDs surface modifications [23]. Meanwhile, targeting delivery of QDs to specific tissues or cells is desirable in image and disease diagnoses. To achieve this goal, QDs are commonly coupled to the surface of a carrier via chemical bonds, and the carrier specifically recognizes some cell surface receptors [24]. Usually, recognizable molecules, such as DNA oligonucleotides [25], RNA [26], peptide [27], antibody [28, 29], receptor ligand [30], and α_v_β_3_ integrin receptor-targeted Arg-Gly-Asp (RGD) polypeptide [31], are introduced on the surface of carriers via chemical conjugation. However, these methods usually are needed to introduce additional targeting groups on the surface of QDs or the carriers. In addition, QDs on the surface of carriers may affect the attachment and entrance into cells of the carrier [32–34].

As a new nano-sized biomaterial, virus-like particles (VLPs) are considered potential biological carriers because of their high similarity in conformation and properties with natural viruses. VLPs contain protein sequences that are related to the binding between virus and cell surface receptors [35, 36]. Thus, VLPs can mimic native virus attachment and entrance to cells, which is favorable for delivery exogenous molecules into specific cells [37–40]. So far, many studies have used VLPs to carry different molecules. For example, murine polyomavirus [41], human papillomavirus [42], hepatitis virus [43], cowpea chlorotic mottle virus [44], simian virus 40 [45], simian vacuolating virus 40 [46], and bacteriophage P22 VLPs [47] had been used to encapsulate magnetic Fe_2_O_3_ nanoparticles, plasmid DNA, and even heterogenous proteins. Research has shown that VLPs from different viruses can improve encapsulated nanomaterials biocompatible. Thus, VLPs are considered a promising nanocapsule for diverse nanomaterials.

**Fig 1:**
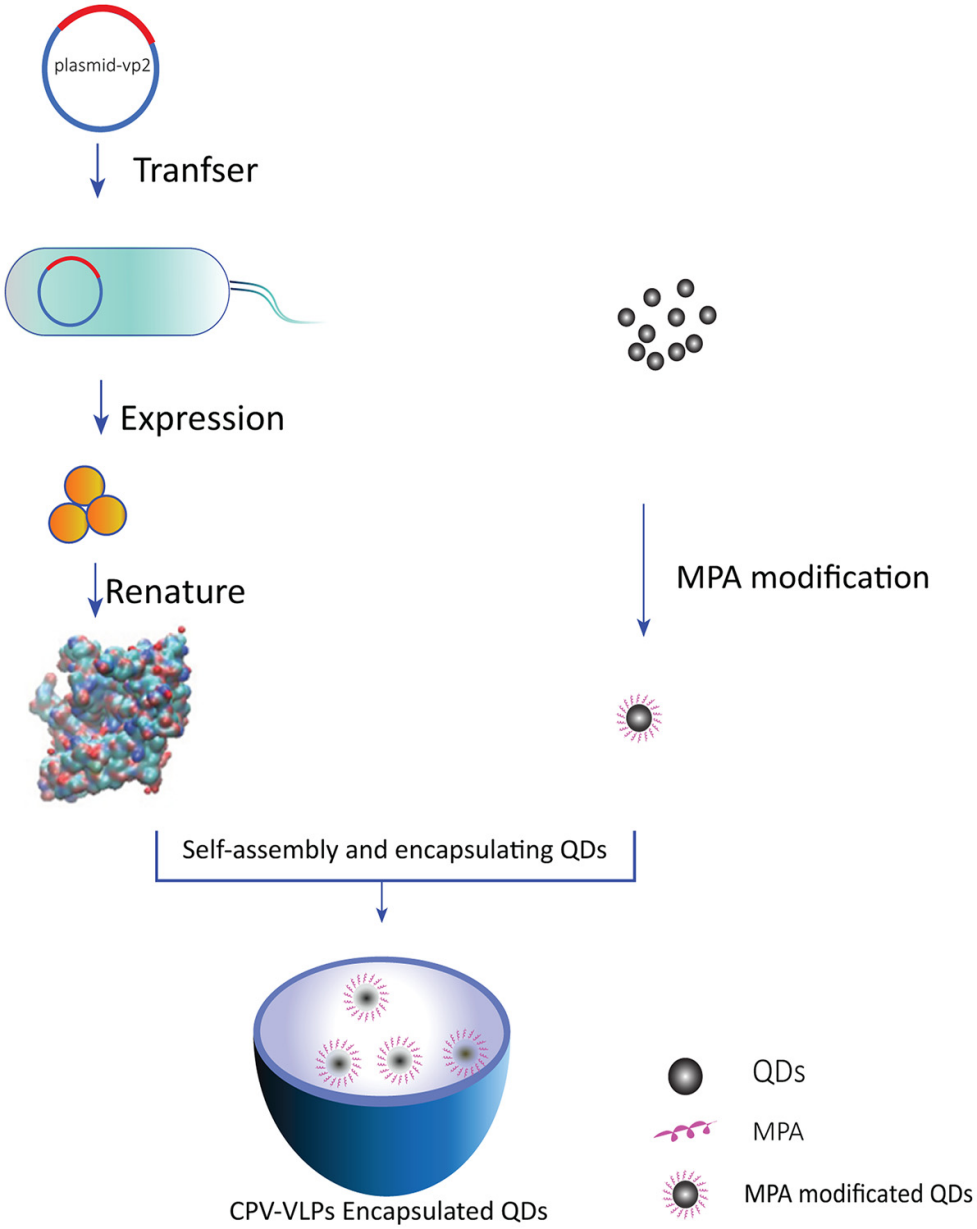
Schematic of encapsulation of QDs with CPV-VLPs. The CPV capsid protein VP2 was expressed in *Escherichia coli*. QDs modified with 3-mercaptopropionic acid (MPA) were then added into VP2 protein solution. During the CPV-VP2 assembly into VLPs, encapsulation of MPA-QDs into VLPs was achieved. The artwork was created using Adobe Illustrator CS6 software.

In our previous experiment, CPV-VLPs were expressed and assembled in an *Escherichia coli* expression system [48]. CPV-VLPs have highly similar morphological structure as that of natural viruses and have almost identical immunologic features with those of natural CPV. Thus, CPV-VLPs can simulate natural virions, and can be specifically absorbed by transferrin receptor-positive cells which native CPV is sensitive. Thus, CPV-VLPs can be used as a transferrin receptor-mediated delivery vehicle [37]. Although previous reports showed that CPV-VLPs obtained by eukaryotic expression systems could be modified and used as a targeted delivery system, no study has demonstrated that CPV-VLPs expressed and assembled in bacterial cells remain the characteristics of a targeting delivery system. In this study, QDs was modified using mercaptopropionic acid (MPA) and encapsulated using CPV-VLPs. QDs is then used in cell labeling experiment to verify that CPV-VLPs as a carrier of QDs can promote biocompatibility and targeting ability for carrying exogenous substances (Fig 1). Our results indicated that CPV-VLPs reduced the toxicity of QDs and enabled to label cells by specific receptor. Therefore, CPV-VLPs have the potential application in biological medicine.

## Materials and Methods

### Cells and Chemical reagents

F81, Hela, and BHK-21 cells, which were purchased from China Center for Type Culture Collection (Wuhan, China), were cultured in minimum essential media containing 10% (v/v) fetal bovine serum and 1% (w/v) penicillin—streptomycin at 37°C and 5% CO_2_.

3-MPA was purchased from Sigma-Aldrich. QDs (shell/core ZnS/CdSe) were a production of Wuhan Jiayuan Quantum Dot Co. LTD (China). 1-Ethyl-3-(3-dimethlyaminopropyl) carbodiimide hydrochloride, N-hydroxysuccinimide were purchased from Sigma-Aldrich. Fluorescein Isothiocyanate (FITC)-labeled goat anti-mouse IgG was purchased from Sigma-Aldrich.

### Expression and assembly of CPV-VLPs

The methods for expressing and self-assembling of CPV-VLPs were elaborated in previous literature [48]. In brief, CPV VP2 gene was subcloned to pSMK vector, which contained a small ubiquitin-like modifier protein (SUMO) and a 6×His tag in the N-terminal fragment of VP2 gene. The plasmid vector was then transformed into *E*. *coli* and induced to express the soluble protein His-SUMO-VP2. Then, fusion protein His-SUMO-VP2 was digested using specific SUMO protease to delete the His-Sumo tag in a dialysis bag. The supernatant containing VP2 protein was collected and assembled overnight at 4°C in a buffer (40 mM Tris-HCl, 500 mM NaCl, 1 mM CaCl_2_, pH 7.0). The CPV-VLPs were then characterized using transmission electron microscopy (TEM) and dynamic light scattering (DLS).

### Modification of QDs by MPA

Water-soluble QDs were prepared as follows: 2 mL of QDs dissolved in n-hexane (initial concentration 8 mmol/mL) was precipitated with 6 mL of ethyl alcohol and centrifuged at 6000 rpm at room temperature for 5 min. The supernatant was discarded, and the sediment was dissolved in 2 mL of chloroform and 1 mL of 3-MPA. The mixture was gently stirred overnight at 4°C, and then was centrifuged at 6000 rpm at room temperature for 5 min. The supernatant was discarded, and the precipitate was rinsed with deionized water and suspended in 2 mL of deionized water. The concentration of water-soluble QDs of a concentration was calculated according to the standard curve.

### Encapsulating MPA-modified QDs by CPV-VLPs

Water-soluble QDs were dispersed using an ultrasound device (Elmasonic E60H) and added into CPV VLPs solution (the concentration is adjusted to about 1 mg/mL) according to different concentration ratios (500:100, 500:200, 500:250, 500:500 v/v). The unencapsulated QDs were removed as followed: The different mixtures were transferred into a 10 kDa dialysis bag, placed in buffer (40 mM Tris-HCl, 150 mM NaCl, 1 mM CaCl_2_, pH 7.4), and mildly stirred overnight at 4°C. Then, the solution in dialysis bag was collected and centrifuged at 12,000rpm, 4°C for 15 min to spin down the CPV-VLPs-QDs. The supernatant in which free QDs existed was discard. The particle size was measured using DLS (Malvern Zeta sizer-Nano ZS90), and the fluorescent absorbance of QDs solution before and after the assembly was detected to calculate the encapsulation rate (EE).

### Determination of release rate of QDs encapsulated in CPV-VLPs

The CPV-VLPs-QDs were transferred into the dialysis bag with 10 kDa molecular weight cut-off, and the bag was then placed in a 500 mL of the PBS buffer (pH 7.0 and 8.0). This system was stirred gently for 72 hours at 4°C. Then, the solution in dialysis bag was collected at 6, 12, 24, 36, 48, and 72 hours, and centrifuged at 12,000rpm, 4°C for 15 min to spin down the CPV-VLPs-QDs. The QDs concentrations in the supernatant were determined by using UV-vis and calculated by following formula. The concentration of QDs in the buffer was detected using UV absorbance at 495 nm.

$$\text{Released QDs}=\frac{\text{Total concentration of QDs }-\text{ The concentration of QDs in the supernatant }}{\text{Total concentration of QDs}}\times 100\text{\%}$$

### MTT **assay**

The cytotoxicity of the CPV-VLPs-QDs and QDs was evaluated using 3-(4, 5-dimethylthiazol-z-yl)-2, 5-diphenyltetrazolium bromide (MTS) assay (Promega). Briefly, F81, Hela, and BHK-21 cells were inoculated in a 96-well plate with 5×10^3^ cells in each well. After 24 hours of normal culture, the cells were treated with CPV-VLPs-QDs and water-soluble QDs (the concentration of QDs in the CPV-VLPs-QDs was consistent with that of water-soluble QDs). After 24 hours of treatment, approximately 20 μL of MTS solution (0.5 mg/mL) was then added into each well. After 3.5 hours, a microplate reader (Bio-Rad Laboratories Inc., USA) was used to detect the absorbance at 495 nm to determine cell viability according to the manufacturer.

### Cellular uptake of CPV-VLPs-QDs

F81, Hela, and BHK-21 cell lines were separately inoculated on a 35 mm culture dishes with slides at a density of 5×10^4^ cells/well. After 24 hours, CPV-VLPs-QDs and water-soluble QDs of equal volume (the concentration of QDs in CPV-VLPs-QDs was consistent with that of water-soluble QDs) were added to each culture dish. The supernatant was discarded, and the cells were rinsed with PBS three times after 1 hour of treatment. The cells were fixed with 4% paraformaldehyde for 15 min at room temperature, penetrated with 1‰ Triton x-100 solution for 15 min at room temperature; Then, the cells were incubated with mouse anti-CPV monoclonal antibody (1:200) for 1 hour at 37°C; and incubated with FITC-labeled goat anti-mouse second antibody (1:500) at 37°C for 1 hour; nuclei were stained with DAPI for 15 min. Each step was followed by rinsing with PBS for 3 times. The slide was observed under 100× objective lens. DAPI, FITC and QDs, were excited at 350, 488, and 561 nm. The Image-Pro plus 6.0 soft was used to measure the fluorescence intensity of FITC and QDs.

## Results

### Expression and assembly of CPV-VLPs

**Fig 2:**
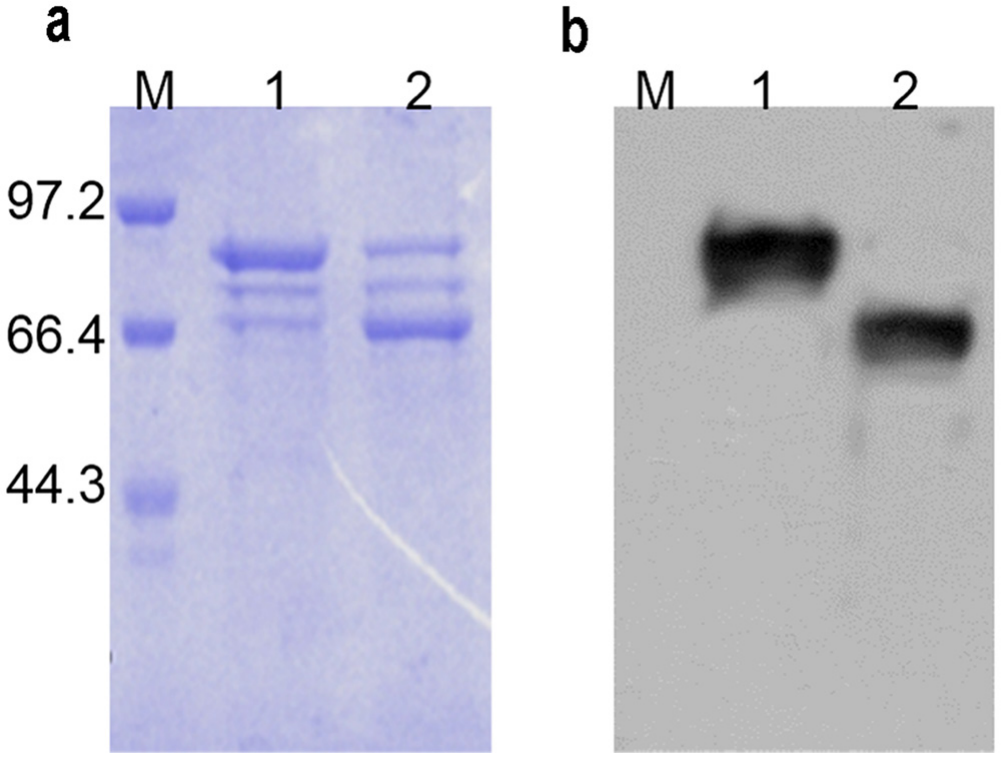
Expression and purification of CPV-VP2 proteins. Protein detection using SDS-PAGE (**a**): lane 1, His-Sumo-VP2 (85 kDa); lane 2, VP2 protein (65 kDa). Identification of proteins using Western blot (**b**): lane 1, His-Sumo-VP2; lane 2, VP2 protein. Mouse anti-CPV monoclonal antibody was used as the primary antibody (1:1000).

To obtain the CPV-VLPs, the expression and purification of the CPV-VP2 proteins that self-assemble into VLPs were performed as described in previous literature [48]. The recombinant CPV-VP2 proteins were verified with SDS-PAGE (Fig 2a) and western blot (Fig 2b) after digestion with or without SUMO-enzyme, respectively. Fig 2 shows that the molecular weight of CPV VP2 protein was approximately 65 kDa, which was consistent with its actual size.

### Modification of QDs using 3-mercaptopropionic acid

**Fig 3:**
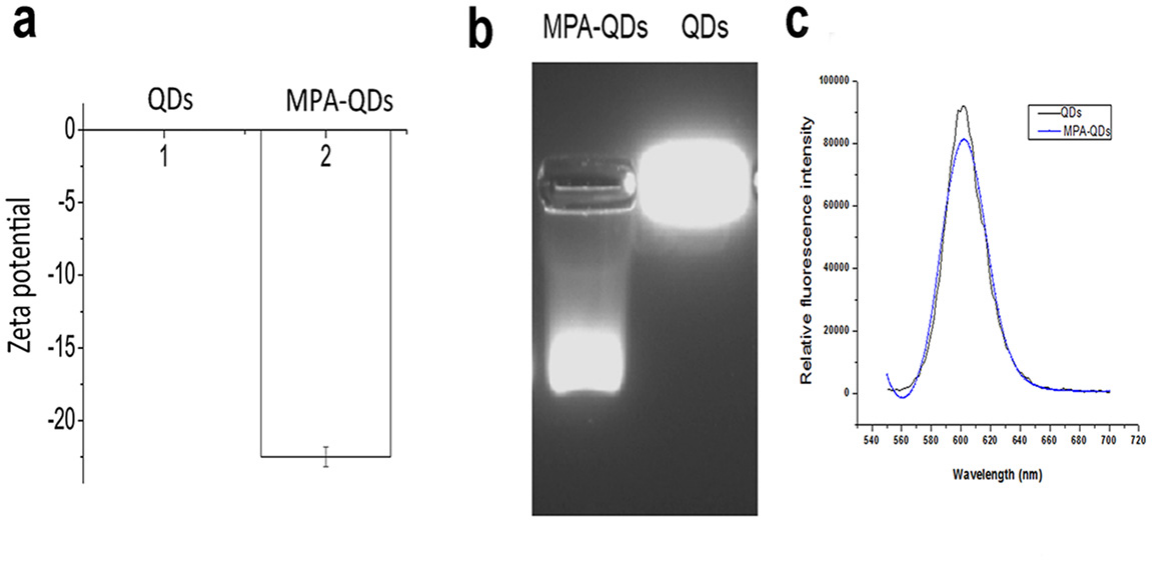
Modification of QDs with MPA. (**a**) Zeta potential of MPA-QDs and QDs. The QDs surface had no charge, but this surface became negative after modification with MPA; (**b**) Gel electrophoresis of MPA-QDs and QDs in the presence of an electric field. QDs without charge stayed only in the well, and MPA-QDs ran to anode. (**c**) Fluorescent emission spectra of MPA-QDs and QDs.

To enhance the water-solublility of QDs, namely, transforming QDs from organic solution into water-soluble solutions, which is challenge for medical application of QDs. The simplest method is surface modification, i.e., adding hydrophilic groups on the molecular surface to form water-soluble QDs with excellent dispersibility [49]. In the present study, MPA was used to modify QDs according to the protocol described in the **Modification of QDs by MPA**. The modified QDs can disperse in the water evenly, and the solution is clear and transparent (data not shown). The initial concentration of the modified QDs was 8 mmol/mL. To confirm that QDs surface was negatively charged after modification with MPA, the MPA-QDs was detected using agarose gel electrophoresis after the verification of charge properties using Malvern Zeta sizer-Nano ZS90 (Fig 3a). Fig 3b shows that the modified QDs moved toward the anode in the electric field, whereas the unmodified QDs did not move and just remained in the sample loading pool. Combined with the result in Fig 3a, MPA modification results in negatively charged surfaces of QDs. To confirm that MPA did not affect the fluorescent property of QDs, UV-vis was used to detect the absorbance of QDs. As shown in Fig 3c, the specific absorbance of QDs is consistent with MPA-modified QDs, which suggested that MPA did not affect the fluorescent property of QDs.

### Optimization of ratio CPV-VLPs to QDs

**Fig 4:**
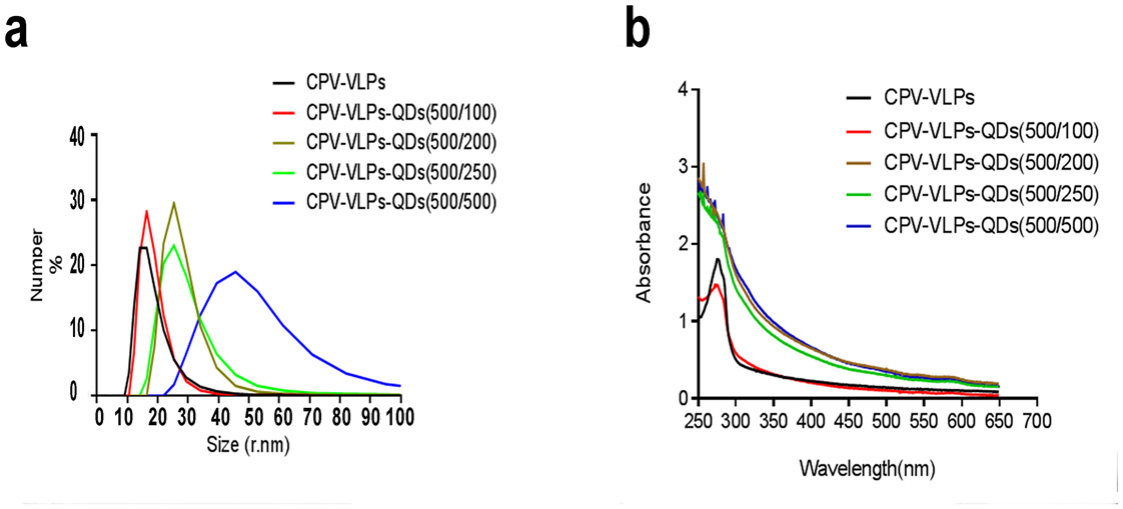
Optimization of QDs encapsulation using CPV-VLPs. (**a**) Particle size of CPV-VLPs-QDs under different CPV-VLPs/QDs ratios. (**b**)Absorbance of complex at different CPV-VLPs/QDs ratios.

To ensure that VLPs encapsulated the QDs, the encapsulation ratio of QDs to CPV-VLPs under different concentrations was explored (Fig 4). As shown in Fig 4a and 4b, the particle size and absorbance property changed with the ratio of VLPs to QDs, which showed that the particle size increased with the quantity of QDs. Similarly, the absorbance of CPV-VLPs-QDs deviated from the specific absorbance curve of QDs. Combined with the results in Fig 4a and 4b, especially the TEM pictures, the ratio of VLPs to QDs 500:100 (v/v) was shown to be optimal. Therefore, the ratio of VLPs to QDs 500:100 was selected as the optimal ratio for all subsequent experiments.

In addition, the EE of QDs in CPV-VLPs was calculated from the QDs concentration. The QDs concentration in complex of ratio 500:100 (v/v) was determined according to the standard curve plotted with the help of known concentrations. EE was calculated using the following formula. Under this ratio of 500:100 (v/v), the EE was approximately 17%; the concentration of QDs in CPV-VLPs-QDs was approximately 1.36 mmol/mL.

$$\text{EE}=\frac{\text{Total concentration of QDs }-\text{ The concentration of QDs in the bag after overnight dialysis}}{\text{CPV}-\text{VP}2\text{ concentration}}\times 100\%$$

### Confirmation of CPV-VLPs-QDs complex

**Fig 5:**
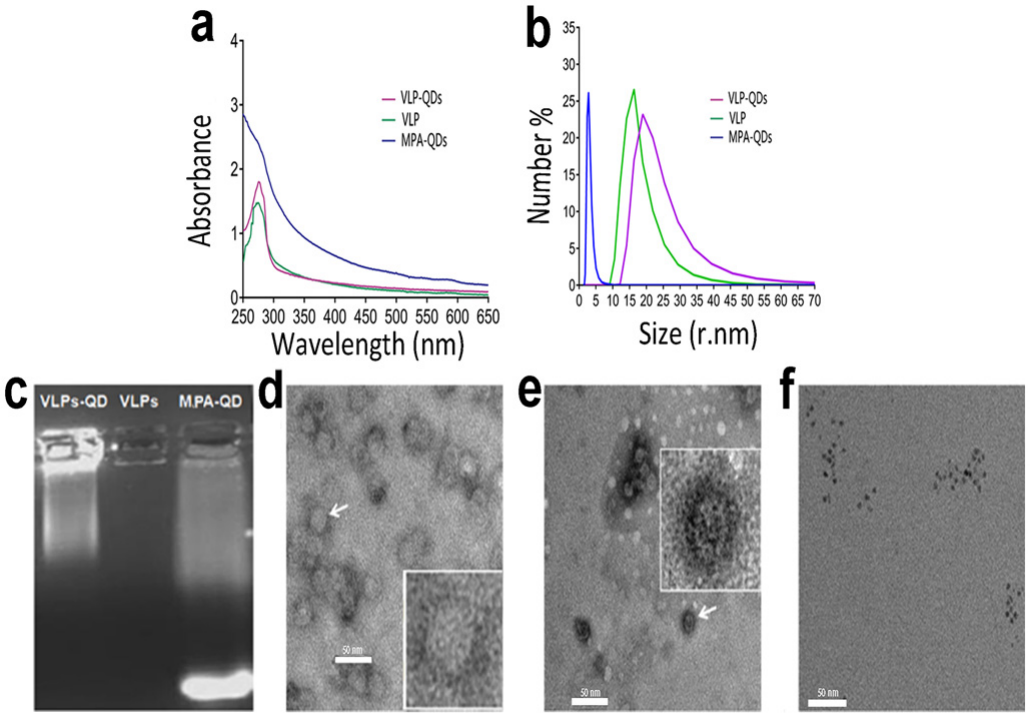
Characterization of CPV-VLPs-QDs complex. (**a**) Absorbance spectrum of CPV-VLPs-QDs complex. (**b**) Particle size of CPV-VLPs-QDs complex using a nano sizer; (**c**) MPA-QDs (blue line) are approximately 5 nm in diameter; the CPV-VLPs (green line) are approximately 20 nm in diameter; the CPV-VLPs-QDs (purple line) are approximately 24 nm in diameter. (**d**) Gel electrophoresis of different nanoparticles. Modified QDs moved toward the anode, whereas the unmodified QDs are still in the well; TEM pictures of CPV-VLPs; (**e**) TEM pictures of CPV-VLPs-QDs complex; **(f)** TEM pictures of soluble MPA-QDs.

To further confirm the encapsulation of QDs in CPV-VLPs, the CPV-VLPs-QDs were detected using fluorescent spectrometry, DLS analyses, gel electrophoresis, and TEM. In Fig 5a, the absorbance of CPV-VLPs-QDs showed a similar peak as that of VLPs, which suggested that VLPs is one of the complex components. Moreover, DLS analyses showed that the particle size of CPV-VLPs is approximately 20 nm, which is similar to that of the native virus. However, the size of CPV-VLPs-QDs is more than 20 nm (Fig 5b), possible explanations for the bigger size of CPV-VLPs-QDs may be that the MPA chains on QDs might stretch the holes of CPV VLPs. As expected, most CPV-VLPs-QDs were still in the wells, but the modified QDs moved toward the anode in the electric field after gel electrophoresis and observation under the UV lamp (Fig 5c). This phenomenon suggests that the QDs were encapsulated into the VLPs and the CPV-VLPs-QDs cannot move as quickly as MPA-QDs, which also confirmed the formation of CPV-VLPs-QDs. To further confirm the CPV-VLPs-QDs visually, the different nanoparticles were observed using TEM. As shown in Fig 5d after digestion through Sumo-specific protease overnight, the VP2 protein was successfully self-assembled in the buffer to form CPV-VLPs, the size of CPV-VLPs is approximately 20 nm which is consistent with the result of DLS. However, the CPV-VLPs-QDs (Fig 5e) is larger than CPV-VLPs in the TEM picture and several MPA-QDs in the VLPs can be observed in the magnified picture. The MPA-modified QDs are shown in Fig 5f. Thus, Fig 5 indicated that VLPs can be assembled and encapsulated the QDs to form the CPV-VLPs-QDs complex.

### Stability of CPV-VLPs-QDs

**Fig 6:**
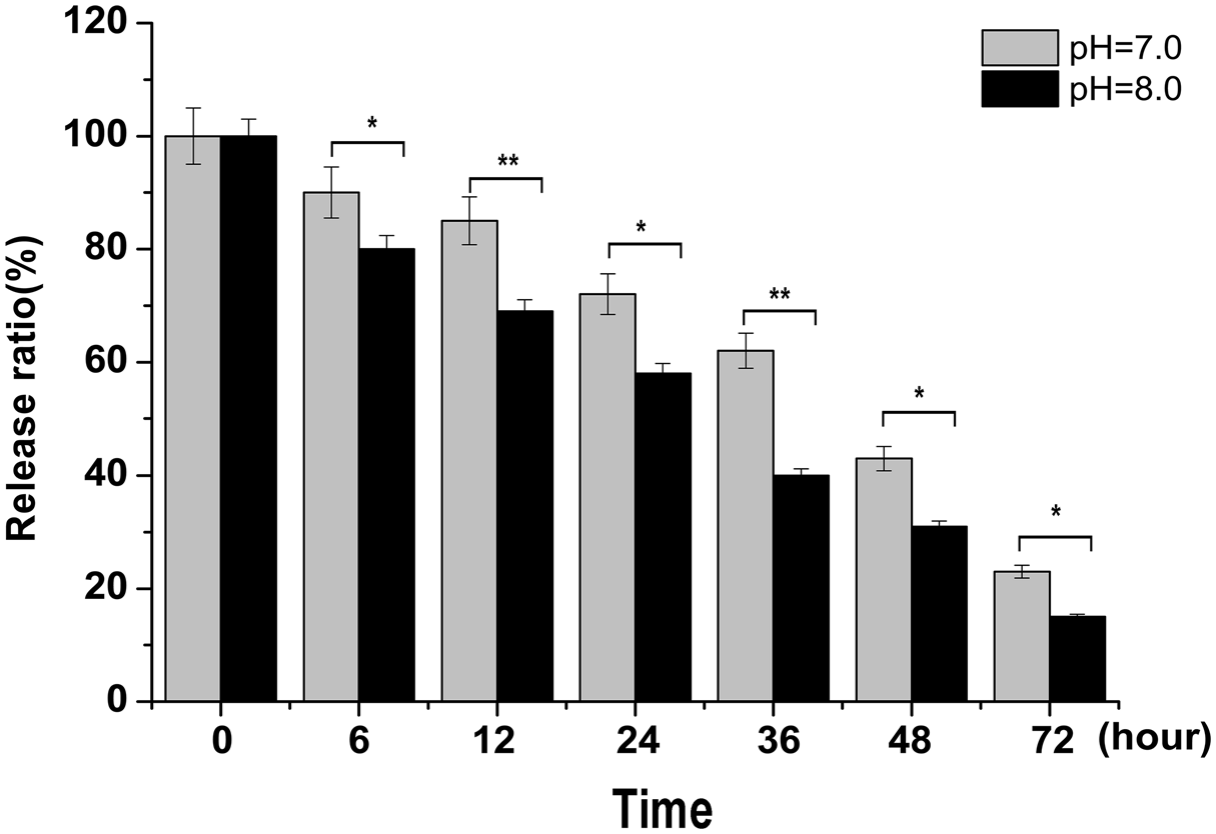
Stability of CPV-VLPs-QDs complex. The CPV-VLPs-QDs were in dialysis under a buffer with different pH values. The QDs concentrations in the buffer were determined at 6, 12, 24, 36, 48, and 72 hours. The released QDs were calculated according to the formula indicated in the **Determination of release rate of QDs encapsulated in CPV-VLPs** of the **Materials and Methods** section. *p value<0.05, **p value<0.01

The stability of CPV-VLPs-QDs in mimic physiological environment was determined by dialysis. CPV-VLPs-QDs were added into the dialysis bag with different pH value (pH 7.0 or pH 8.0) at 4°C. The fluorescence intensity was detected at different times of dialysis. Release kinetics curve of the QDs was calculated based on the standard curve. The initial concentration of QDs minus the concentration of QDs in the buffer at different time points is the concentration of QDs in CPV-VLPs-QDs. As shown in Fig 6, the complex was relatively stable and only 50% of QDs were released after approximately 48 hours under a neutral environment. However, the QDs in weak alkaline solution was released quicker, compared with CPV-VLPs-QDs in neutral solution, which indicates that the CPV-VLPs-QDs complex is not stable in alkaline environment, but could be stable in normal physiological environment.

### Cytotoxicity of CPV-VLPs-QDs

**Fig 7:**
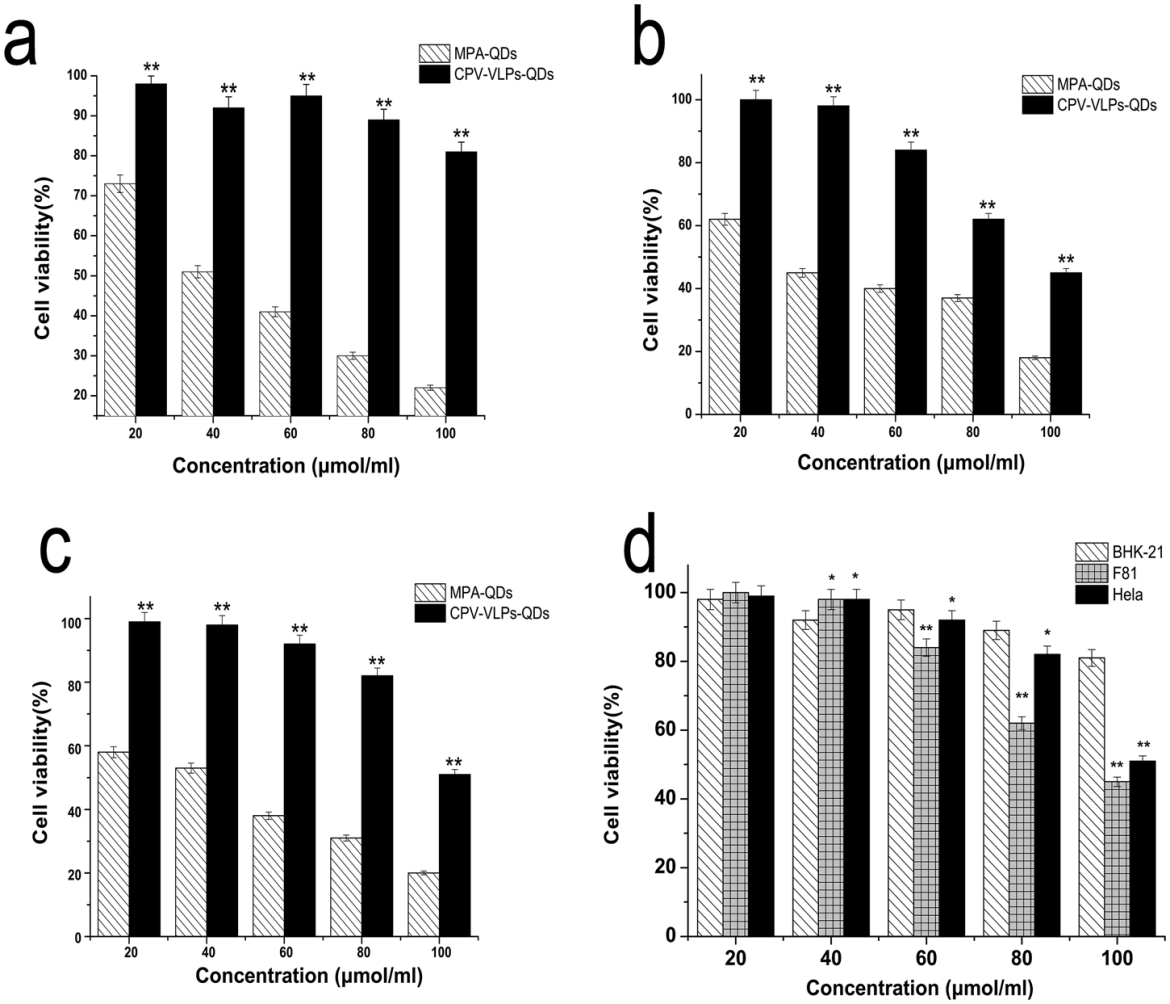
Cytotoxicity of the CPV-VLPs-QDs and MPA-QDs in BHK-21(a), F81 (b), and Hela (c) cells. The relative cell viability (%) related to control wells containing cell culture medium without nanoparticles was calculated as [*A*]_sample_ /[*A*]_control_ × 100%, where [*A*]_Sample_ is the absorbance of the test sample and [A]_control_ is the absorbance of control sample. To determine 50% inhibitory concentration, namely, IC50, concentration—response curves were generated relative to the negative control. IC50 values were calculated from the non-linear regression analyses. In comparison with MPA-QDs, the encapsulated QDs, i.e., CPV-VLPs-QDs with low cytotoxicity (**d**), show that CPV-VLPs can be employed as safe delivery carriers. *p value<0.05, **p value<0.01.

To determine whether the VLPs decreased the cytotoxicity of QDs, the CPV-VLPs-QDs complex (at 20, 40, 60, 80, and 100 μmol/mL) was added in the F81, Hela, and BHK-21 cells, respectively. Cell viability was detected using MTS solution after a 24 hours treatment with MPA-QDs and CPV-VLPs-QDs complex. As shown in Fig 7, MPA-QDs had higher cytotoxicity on three cell lines compared with CPV-VLPs-QDs. The MPA-QDs IC50 values were 49, 40, and 41 μmol/mL against BHK-21, F81, and Hela cells, respectively. The CPV-VLPs-QDs IC50 values were 282, 98, and 122 μmol/mL against BHK-21, F81, and Hela cells (Microcal Origin 8.5 software). The cell viability of BHK-21 decreased more slowly than those of the F81 or Hela cells that were treated with CPV-VLPs-QDs. When the concentration exceeded a threshold for imaging (60 μmol/mL), the cytotoxicity of CPV-VLPs-QDs on F81 and Hela cells was stronger than that on BHK-21 cells. Previous studies indicate that CPV-VLPs have a strong capacity in targeting the transferrin receptor-positive cells [50]. Therefore, these studies established that F81 and Hela cells could absorb more CPV-VLPs-QDs, and the cytotoxicity of QDs could have main functions in cell viability.

### Cell-targeted labeling

To assess whether the CPV VLPs encapsulating QDs still preserved their activity in entering cells with transferrin receptor, the hybrid VLPs-QDs were allowed to incubate with different cells, including F81, Hela and BHK-21 cells. F81 cell is the host cell line of CPV infection, but the BHK-21 cell is not sensitive for the infection of CPV (S1 and S2 Figs). Hela cell was selected because of its high level expression of transferrin receptor.

**Fig 8:**
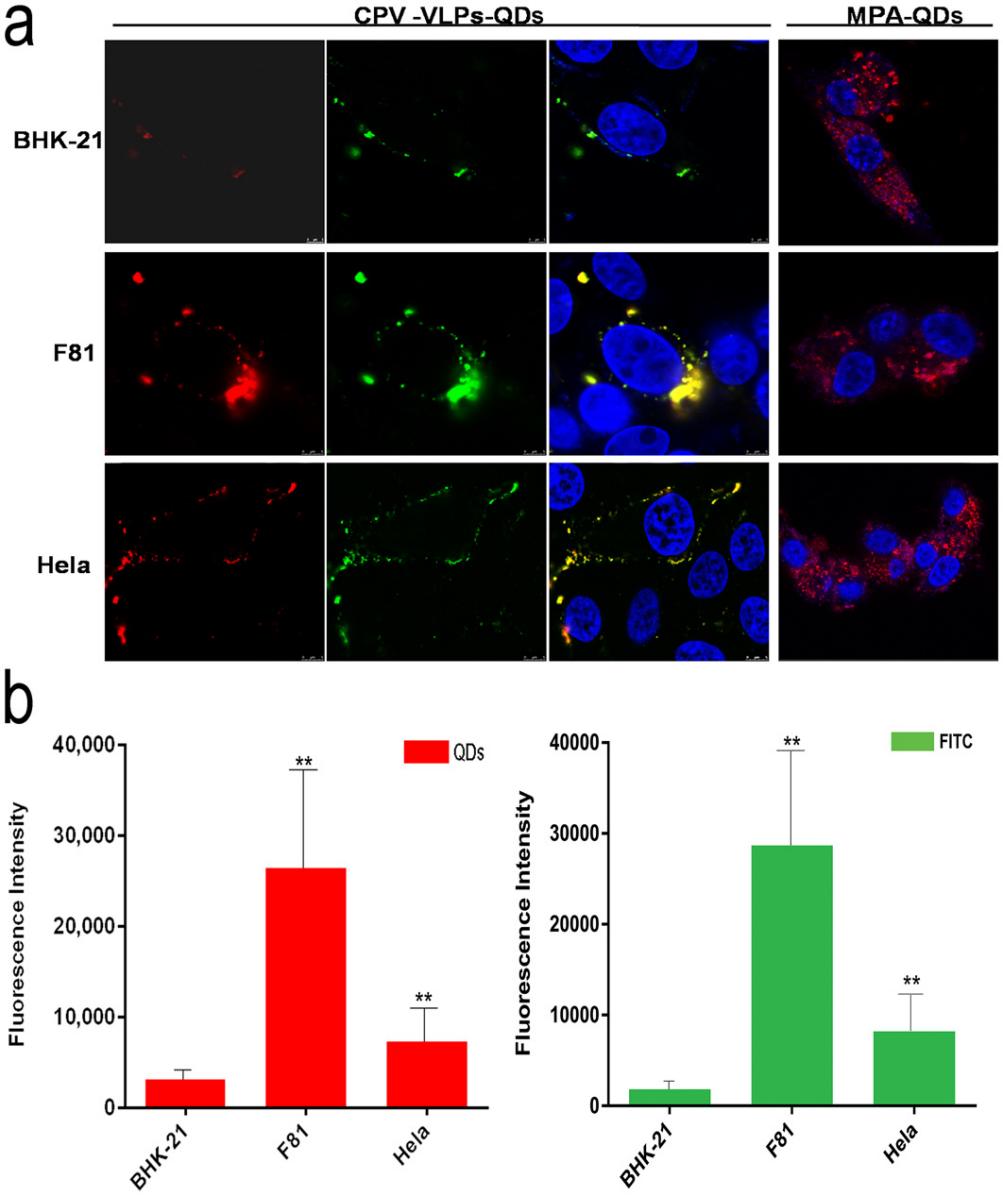
Targeting labeling of cells using CPV-VLPs-QDs. DAPI-labeled nucleus (blue), QDs (red), and FITC-labeled goat anti-mouse secondary antibody (green). Anti-CPV mouse monoclonal antibody was used as the primary antibody. Hela and F81 cells show high uptake (red), whereas no obvious fluorescence signal was detected in BHK-21 cells.

As shown in Fig 8a, BHK-21 cells yielded little fluorescence signal. Differently, all CPV VLPs-encapsulated QDs bound to F81 cells and entered efficiently. It suggested that encapsulation enhanced the biological effects of QDs deriving from CPV VLPs. The same results were observed for Hela cells. On the contrary, QDs coated with MPA easily entered randomly to the cell. In addition, the fluorescent intensity of QDs and FITC were analyzed by Image-pro plus 6.0 software (Fig 8b). The specific fluorescent intensity of QDs and FITC in F81 cells are highest than that of Hela and BHK-21 cells. These observations indicate that CPV VLPs maintain the ability to enter transferrin receptor-positive cells.

## Discussion

In this study, CPV-VLPs that assembled using VP2 proteins were used to encapsulate QDs in vitro. The formation of CPV-VLPs-QDs is correlated with many factors. Therefore, the CPV-VLPs-QDs complex was detected at different ratios of VLPs to QDs to maximize the encapsulation efficiency of MPA-QDs. With the increase of QDs concentration, the particle size of CPV-VLPs-QDs gradually increased. When the ratio of VLPs:QDs exceeded 500:500 (v/v), and the particle size increased significantly because of VP2 protein aggregation. The optimal concentration ratio of 500:100 (VLPs: QDs, v/v) was adopted, under which the concentration of encapsulated QDs was approximately 1.36 mmol/mL.

The stability test in vitro indicated that the CPV-VLPs-QDs were stable in the condition similar to in cells, which is consistent with other VLPs, such as cowpea mosaic virus, SV40, CCMV, brome mosaic virus, and MS2. In addition, cytotoxicity of CPV-VLPs-QDs showed that CPV-VLPs significantly decreased the toxicity of QDs particles, which confirmed the high biocompatibility of CPV-VLPs and extended the application field of QDs. Therefore, CPV-VLPs-QDs may be utilized for the imaging of live cells, as well as in vivo imaging, the present study indicated that encapsulation using CPV-VLPs reduces the toxic effects of QDs, which could be attributed to the fact that encapsulation prevented QDs from having direct contact to cells and thereby delaying the release of toxic ions from QDs.

In addition, previous experiments confirmed that CPV-VLPs can enter cells via the same pathway as that of the native CPV virion; the major pathway is receptor-mediated cell endocytosis [37–39]. Encapsulation using CPV-VLPs not only resulted in increasing the single QDs biocompatibility but also improved the targeted ability with respect to specific recognition of transferrin receptors. In this study, we have designed and evaluated a method that could encapsulate QDs into CPV-VLPs in vitro. Although further improvement may be needed, the approach of QDs-encapsulation established may facilitate the application of QDs in biological imaging, especially in the targeted imaging of cancer diagnosis. This encapsulation method promotes the feasibility of QDs as receptor-targeted nanoparticles in biological medical field. Moreover, other hydrophobic drugs (paclitaxel), inorganic particles (e.g., QDs), and magnetic particles could also be effectively encapsulated in the internal cavities of CPV-VLPs, which will widen the biological application of CPV-VLPs as carriers.

In conclusion, the CPV-VLPs assembled by CPV-VP2 capsid protein, which expressed in different expression systems can be used to achieve tumor-specific delivery by natural affinity of transferrin receptors [37, 39, 51]. Thus, the CPV-VLPs can be a candidate to modify different nanoparticles and achieve the targeting delivery.

## Supporting Information

S1 Text Supporting caption of S1 Fig. (DOCX)

S2 Text Supporting caption of S2 Fig. (DOCX)

S1 Fig Fluorescent image of CPV VLPs in cells. (TIF)

S2 Fig Fluorescent image and Quantitative analysis of fluorescence intensity of infectious virus in different cells. (TIF)

## References

[1] Zrazhevskiy P, Sena M, Gao X. Designing multifunctional quantum dots for bioimaging, detection, and drug delivery. Chemical Society reviews. 2010;39(11):4326–54. 10.1039/b915139g 20697629 PMC3212036

[2] Xu J, Ruchala P, Ebenstain Y, Li JJ, Weiss S. Stable, compact, bright biofunctional quantum dots with improved peptide coating. The journal of physical chemistry B. 2012;116(36):11370–8. Epub 2012/08/21. 10.1021/jp306453y 22900542 PMC3470653

[3] Liu JH, Yang ST, Chen XX, Wang H. Fluorescent carbon dots and nanodiamonds for biological imaging: preparation, application, pharmacokinetics and toxicity. Current drug metabolism. 2012;13(8):1046–56. . 22380012 10.2174/138920012802850083

[4] Kairdolf BA, Smith AM, Stokes TH, Wang MD, Young AN, Nie S. Semiconductor quantum dots for bioimaging and biodiagnostic applications. Annual review of analytical chemistry (Palo Alto, Calif). 2013;6:143–62. Epub 2013/03/27. 10.1146/annurev-anchem-060908-155136 23527547 PMC3733675

[5] Pinaud F, Clarke S, Sittner A, Dahan M. Probing cellular events, one quantum dot at a time. Nat Methods. 2010;7(4):275–85. 10.1038/nmeth.1444 . 20354518

[6] Zhou W, Baneyx F. Aqueous, protein-driven synthesis of transition metal-doped ZnS immuno-quantum dots. ACS nano. 2011;5(10):8013–8. Epub 2011/09/29. 10.1021/nn2024896 21942544 PMC3204801

[7] Gao J, Chen X, Cheng Z. Near-infrared quantum dots as optical probes for tumor imaging. Current topics in medicinal chemistry. 2010;10(12):1147–57. Epub 2010/04/15. 20388111 10.2174/156802610791384162 PMC3629965

[8] Shao L, Gao Y, Yan F. Semiconductor quantum dots for biomedicial applications. Sensors (Basel, Switzerland). 2011;11(12):11736–51. Epub 2012/01/17. 10.3390/s111211736 22247690 PMC3252007

[9] Byers RJ, Hitchman ER. Quantum dots brighten biological imaging. Progress in histochemistry and cytochemistry. 2011;45(4):201–37. 10.1016/j.proghi.2010.11.001 . 21196026

[10] Geszke-Moritz M, Moritz M. Quantum dots as versatile probes in medical sciences: synthesis, modification and properties. Materials science & engineering C, Materials for biological applications. 2013;33(3):1008–21. 10.1016/j.msec.2013.01.003 . 23827537

[11] Valizadeh A, Mikaeili H, Samiei M, Farkhani SM, Zarghami N, Kouhi M, et al Quantum dots: synthesis, bioapplications, and toxicity. Nanoscale research letters. 2012;7(1):480 10.1186/1556-276X-7-480 22929008 PMC3463453

[12] Rzigalinski BA, Strobl JS. Cadmium-containing nanoparticles: perspectives on pharmacology and toxicology of quantum dots. Toxicology and applied pharmacology. 2009;238(3):280–8. Epub 2009/04/22. 10.1016/j.taap.2009.04.010 19379767 PMC2709954

[13] Bottrill M, Green M. Some aspects of quantum dot toxicity. Chemical communications (Cambridge, England). 2011;47(25):7039–50. 10.1039/c1cc10692a . 21475767

[14] Ye F, Barrefelt A, Asem H, Abedi-Valugerdi M, El-Serafi I, Saghafian M, et al Biodegradable polymeric vesicles containing magnetic nanoparticles, quantum dots and anticancer drugs for drug delivery and imaging. Biomaterials. 2014;35(12):3885–94. 10.1016/j.biomaterials.2014.01.041 . 24495486

[15] Santra S. The potential clinical impact of quantum dots. Nanomedicine (London, England). 2012;7(5):623–6. Epub 2012/05/29. 10.2217/nnm.12.45 . 22630145

[16] Ho YP, Leong KW. Quantum dot-based theranostics. Nanoscale. 2010;2(1):60–8. Epub 2010/07/22. 10.1039/b9nr00178f 20648364 PMC2965651

[17] Anderson RE, Chan WC. Systematic investigation of preparing biocompatible, single, and small ZnS-Capped CdSe quantum dots with amphiphilic polymers. ACS nano. 2008;2(7):1341–52. Epub 2009/02/12. 10.1021/nn700450g . 19206301

[18] Tekle C, Deurs B, Sandvig K, Iversen TG. Cellular trafficking of quantum dot-ligand bioconjugates and their induction of changes in normal routing of unconjugated ligands. Nano letters. 2008;8(7):1858–65. Epub 2008/06/24. 10.1021/nl0803848 . 18570482

[19] Mukthavaram R, Wrasidlo W, Hall D, Kesari S, Makale M. Assembly and targeting of liposomal nanoparticles encapsulating quantum dots. Bioconjugate chemistry. 2011;22(8):1638–44. 10.1021/bc200201e 21786821 PMC3160765

[20] Tan WB, Zhang Y. Surface modification of gold and quantum dot nanoparticles with chitosan for bioapplications. Journal of biomedical materials research Part A. 2005;75(1):56–62. 10.1002/jbm.a.30410 . 16086419

[21] Adamczak M, Hoel HJ, Gaudernack G, Barbasz J, Szczepanowicz K, Warszynski P. Polyelectrolyte multilayer capsules with quantum dots for biomedical applications. Colloids and surfaces B, Biointerfaces. 2012;90:211–6. 10.1016/j.colsurfb.2011.10.028 . 22078925

[22] Shi Y, Liu L, Pang H, Zhou H, Zhang G, Ou Y, et al Facile preparation of highly luminescent CdTe quantum dots within hyperbranched poly(amidoamine)s and their application in bio-imaging. Nanoscale research letters. 2014;9(1):115 Epub 2014/03/15. 10.1186/1556-276X-9-115 24624925 PMC4007777

[23] Lee YK, Jeong JM, Hoigebazar L, Yang BY, Lee YS, Lee BC, et al Nanoparticles modified by encapsulation of ligands with a long alkyl chain to affect multispecific and multimodal imaging. Journal of nuclear medicine: official publication, Society of Nuclear Medicine. 2012;53(9):1462–70. Epub 2012/08/04. 10.2967/jnumed.111.092759 . 22859859

[24] Kaji N, Tokeshi M, Baba Y. Quantum dots for single bio-molecule imaging. Analytical sciences: the international journal of the Japan Society for Analytical Chemistry. 2007;23(1):21–4. . 17213618 10.2116/analsci.23.21

[25] Charoenphol P, Bermudez H. Design and application of multifunctional DNA nanocarriers for therapeutic delivery. Acta biomaterialia. 2013 Epub 2013/07/31. 10.1016/j.actbio.2013.07.021 . 23896566 PMC3874082

[26] Ren S, Kang MR, Wang J, Huang V, Place RF, Sun Y, et al Targeted induction of endogenous NKX3-1 by small activating RNA inhibits prostate tumor growth. The Prostate. 2013;73(14):1591–601. 10.1002/pros.22709 . 23836514

[27] Delehanty JB, Medintz IL, Pons T, Brunel FM, Dawson PE, Mattoussi H. Self-assembled quantum dot-peptide bioconjugates for selective intracellular delivery. Bioconjugate chemistry. 2006;17(4):920–7. Epub 2006/07/20. 10.1021/bc060044i 16848398 PMC2519024

[28] Yin S, Yang S, Shang Y, Sun S, Zhou G, Jin Y, et al Characterization of Asia 1 sdAb from camels bactrianus (C. bactrianus) and conjugation with quantum dots for imaging FMDV in BHK-21 cells. PloS one. 2013;8(5):e63500 Epub 2013/06/06. 10.1371/journal.pone.0063500 23737944 PMC3667858

[29] Veeranarayanan S, Poulose AC, Mohamed MS, Nagaoka Y, Iwai S, Nakagame Y, et al Synthesis and application of luminescent single CdS quantum dot encapsulated silica nanoparticles directed for precision optical bioimaging. International journal of nanomedicine. 2012;7:3769–86. Epub 2012/08/14. 10.2147/IJN.S31310 22888233 PMC3414225

[30] Howarth M, Liu W, Puthenveetil S, Zheng Y, Marshall LF, Schmidt MM, et al Monovalent, reduced-size quantum dots for imaging receptors on living cells. Nat Methods. 2008;5(5):397–9. Epub 2008/04/22. 10.1038/nmeth.1206 18425138 PMC2637151

[31] Zhang Y, Hong G, Zhang Y, Chen G, Li F, Dai H, et al Ag2S quantum dot: a bright and biocompatible fluorescent nanoprobe in the second near-infrared window. ACS nano. 2012;6(5):3695–702. Epub 2012/04/21. 10.1021/nn301218z 22515909 PMC3358570

[32] Joo KI, Lei Y, Lee CL, Lo J, Xie J, Hamm-Alvarez SF, et al Site-specific labeling of enveloped viruses with quantum dots for single virus tracking. ACS nano. 2008;2(8):1553–62. Epub 2008/12/17. 10.1021/nn8002136 19079775 PMC2600658

[33] Zhu Y, Wang F, Zhang C, Du J. Preparation and mechanism insight of nuclear envelope-like polymer vesicles for facile loading of biomacromolecules and enhanced biocatalytic activity. ACS nano. 2014;8(7):6644–54. 10.1021/nn502386j . 24930816

[34] Wen L, Lin Y, Zheng ZH, Zhang ZL, Zhang LJ, Wang LY, et al Labeling the nucleocapsid of enveloped baculovirus with quantum dots for single-virus tracking. Biomaterials. 2014;35(7):2295–301. 10.1016/j.biomaterials.2013.11.069 . 24360719

[35] Lockney DM, Guenther RN, Loo L, Overton W, Antonelli R, Clark J, et al The Red clover necrotic mosaic virus capsid as a multifunctional cell targeting plant viral nanoparticle. Bioconjugate chemistry. 2011;22(1):67–73. 10.1021/bc100361z . 21126069

[36] Zhao Q, Chen W, Chen Y, Zhang L, Zhang J, Zhang Z. Self-assembled virus-like particles from rotavirus structural protein VP6 for targeted drug delivery. Bioconjugate chemistry. 2011;22(3):346–52. Epub 2011/02/23. 10.1021/bc1002532 . 21338097

[37] Singh P, Destito G, Schneemann A, Manchester M. Canine parvovirus-like particles, a novel nanomaterial for tumor targeting. Journal of nanobiotechnology. 2006;4:2 Epub 2006/02/16. 10.1186/1477-3155-4-2 16476163 PMC1386698

[38] Daniels TR, Delgado T, Helguera G, Penichet ML. The transferrin receptor part II: targeted delivery of therapeutic agents into cancer cells. Clinical immunology (Orlando, Fla). 2006;121(2):159–76. Epub 2006/08/22. 10.1016/j.clim.2006.06.006 . 16920030

[39] Singh P. Tumor Targeting Using Canine Parvovirus Nanoparticles. Curr Top Microbiol. 2009;327:123–41. WOS:000261282200006. 10.1007/978-3-540-69379-6_6 19198573

[40] Pokorski JK, Hovlid ML, Finn MG. Cell targeting with hybrid Qβ virus-like particles displaying epidermal growth factor. Chembiochem: a European journal of chemical biology. 2011;12(16):2441–7. 10.1002/cbic.201100469 21956837 PMC3410710

[41] Lipin DI, Chuan YP, Lua LH, Middelberg AP. Encapsulation of DNA and non-viral protein changes the structure of murine polyomavirus virus-like particles. Archives of virology. 2008;153(11):2027–39. Epub 2008/11/04. 10.1007/s00705-008-0220-9 . 18979257

[42] Sanchez-Rodriguez SP, Munch-Anguiano L, Echeverria O, Vazquez-Nin G, Mora-Pale M, Dordick JS, et al Human parvovirus B19 virus-like particles: In vitro assembly and stability. Biochimie. 2012;94(3):870–8. Epub 2011/12/24. 10.1016/j.biochi.2011.12.006 . 22192916

[43] He L, Porterfield Z, van der Schoot P, Zlotnick A, Dragnea B. Hepatitis virus capsid polymorph stability depends on encapsulated cargo size. ACS nano. 2013;7(10):8447–54. 10.1021/nn4017839 . 24010404 PMC5683388

[44] Brasch M, de la Escosura A, Ma Y, Uetrecht C, Heck AJ, Torres T, et al Encapsulation of phthalocyanine supramolecular stacks into virus-like particles. Journal of the American Chemical Society. 2011;133(18):6878–81. 10.1021/ja110752u . 21506537

[45] Li F, Zhang ZP, Peng J, Cui ZQ, Pang DW, Li K, et al Imaging viral behavior in Mammalian cells with self-assembled capsid-quantum-dot hybrid particles. Small (Weinheim an der Bergstrasse, Germany). 2009;5(6):718–26. 10.1002/smll.200801303 . 19242943

[46] Wang T, Zhang Z, Gao D, Li F, Wei H, Liang X, et al Encapsulation of gold nanoparticles by simian virus 40 capsids. Nanoscale. 2011;3(10):4275–82. Epub 2011/09/01. 10.1039/c1nr10568j . 21879117

[47] Lucon J, Qazi S, Uchida M, Bedwell GJ, LaFrance B, Prevelige PE Jr, et al Use of the interior cavity of the P22 capsid for site-specific initiation of atom-transfer radical polymerization with high-density cargo loading. Nature chemistry. 2012;4(10):781–8. Epub 2012/09/25. 10.1038/nchem.1442 23000990 PMC3763733

[48] Xu J, Guo HC, Wei YQ, Dong H, Han SC, Ao D, et al Self-assembly of virus-like particles of canine parvovirus capsid protein expressed from Escherichia coli and application as virus-like particle vaccine. Applied microbiology and biotechnology. 2014;98(8):3529–38. 10.1007/s00253-013-5485-6 . 24413974

[49] Dubertret B, Skourides P, Norris DJ, Noireaux V, Brivanlou AH, Libchaber A. In vivo imaging of quantum dots encapsulated in phospholipid micelles. Science. 2002;298(1095–9203 (Electronic)):1759–62. 10.1126/science.1077194 . 12459582

[50] Li H, Qian ZM. Transferrin/transferrin receptor-mediated drug delivery. Medicinal research reviews. 2002;22(3):225–50. Epub 2002/04/05. . 11933019 10.1002/med.10008

[51] Qian ZM, Li H, Sun H, Ho K. Targeted drug delivery via the transferrin receptor-mediated endocytosis pathway. Pharmacol Rev. 2002;54(4):561–87. . 12429868 10.1124/pr.54.4.561

