# Supplementary material for: Quantum Dots Encapsulated with Canine Parvovirus-Like Particles Improving the Cellular Targeted Labeling
Source: PLoS One. 2015 Sep 23;10(9):e0138883. doi: 10.1371/journal.pone.0138883 (PMC4580430; doi:10.1371/journal.pone.0138883)
Supplement: S1 Text — (DOCX) [file pone.0138883.s001.docx]

**SupPORTING TEXT**

**Title: “*Quantum Dots Encapsulated with Canine Parvovirus-like Particles Improving the Cellular Targeted Labeling*”**

Dan Yan^1^^¶^, Bin Wang ^1,2¶^, Shiqi Sun^1*^, Xia Feng^1^, Ye Jin^1^, Xueping Yao^2^, Suizhong Cao^2^, Huichen Guo^1*^

^1^State Key Laboratory of Veterinary Etiological Biology and National Foot and Mouth Disease Reference Laboratory, Lanzhou Veterinary Research Institute, Chinese Academy of Agricultural Sciences, Xujiaping 1, Lanzhou, Gansu 730046, China

^2^College of Veterinary Medicine, Sichuan Agricultural University, Chengdu, Sichuan, 611130, China

^¶^Both of authors contributed equally to this work.

**Corresponding authors:** A/Prof. Shiqi Sun; Prof. Huichen Guo

State Key Laboratory of Veterinary Etiological Biology and National Foot and Mouth Disease Reference Laboratory, Lanzhou Veterinary Research Institute, Chinese Academy of Agricultural Sciences,

Xujiaping 1, Lanzhou, Gansu, 730046, P.R China.

**Corresponding authors’** **E-mail:** [sunshiqi@caas.cn](mailto:sunshiqi@caas.cn); ghch-2004@hotmail.com.

**S1. Cell specific binding and internalization of CPV-VLPs.**

The processes were following: F81, BHK-21 and Hela cells about 10,000 cells/well were plated in a 24-well tissue culture plate containing circular glass cover slips, after 24 hours incubation, the cells were exposed to 0.5 mg/ml of CPV-VLPs 2 hours at 37℃ in media. Following incubation the cells were washed 3 times with cold PBS and then fixed with 4% paraformaldehyde in for 15 min. After fixing, the cells were washed 3 times with PBS and then treated for 15 min 0.1% Triton X-100 in PBS. The cells were then exposed to with mouse anti anti-CPV monoclonal antibody (1:200) for 1 hour at 37 °C diluted in 1% new born calf serum (NBS) for 1 hour at room temperature. The cells were washed three times in PBS and exposed to FITC-labeled 1:500 goat anti-mouse antibodies (Sigma, US) in 1% NBS for 1 hour at room temperature. The cover slips were washed three times with PBS. Lastly the cells were treated with DAPI (Sigma) for nuclear staining. Following washing with PBS three times, the cells were directly examined with a Lecia confocal microscope.
