# Supplementary figures and images for: Quantum Dots Encapsulated with Canine Parvovirus-Like Particles Improving the Cellular Targeted Labeling
Source: PLoS One. 2015 Sep 23;10(9):e0138883. doi: 10.1371/journal.pone.0138883 (PMC4580430; doi:10.1371/journal.pone.0138883)

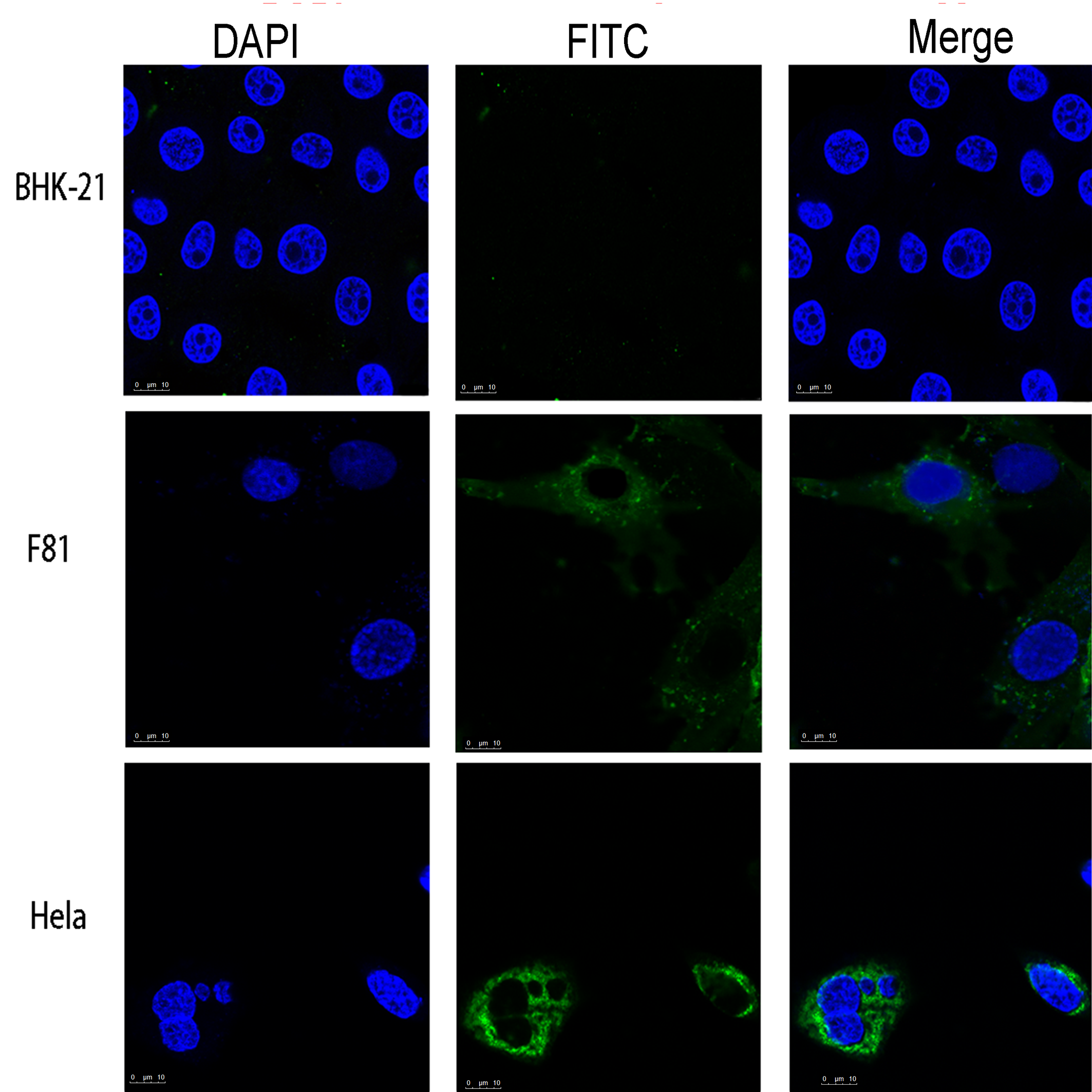

Supplement: S1 Fig — (TIF) [file pone.0138883.s003.tif]

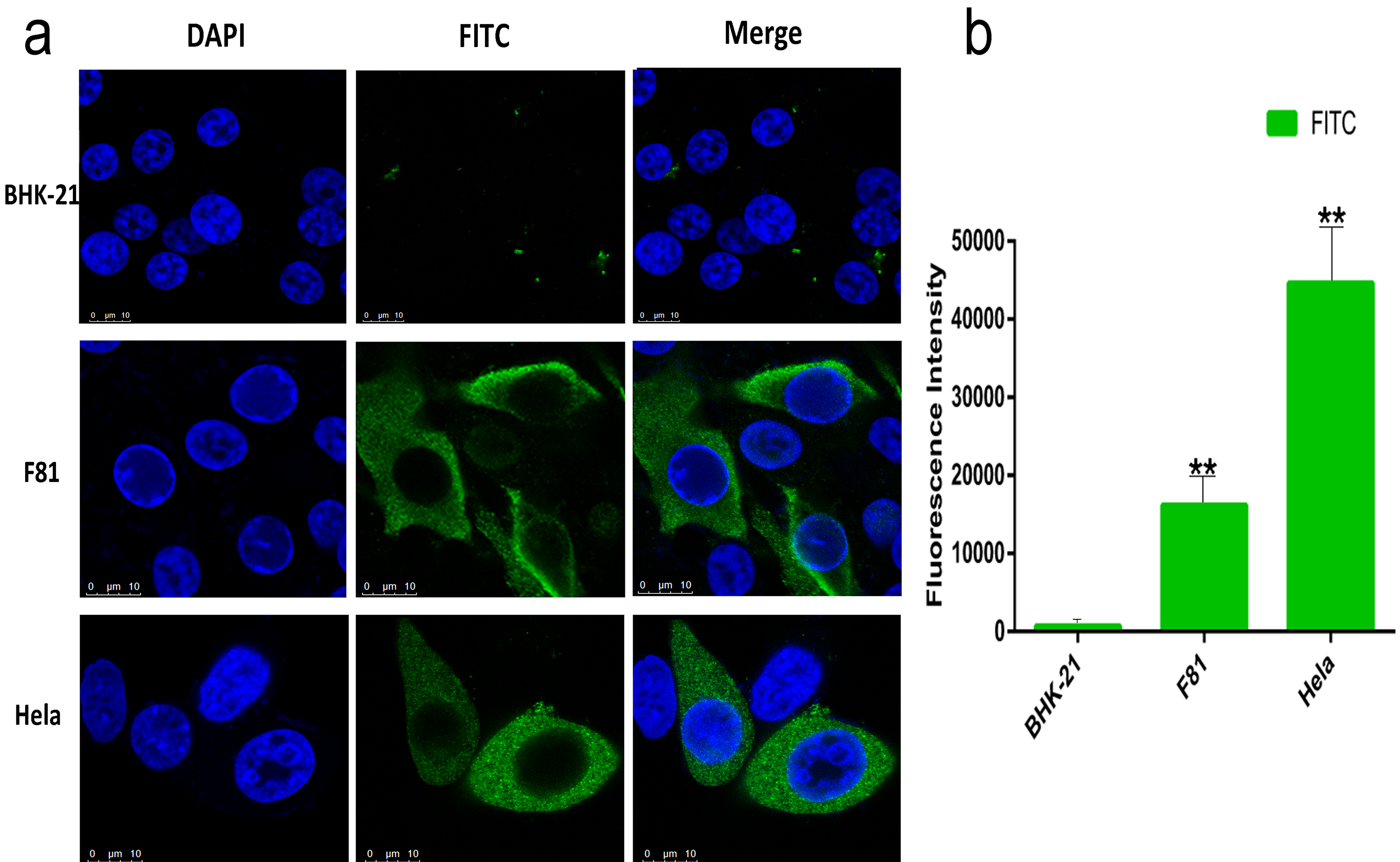

Supplement: S2 Fig — (TIF) [file pone.0138883.s004.tif]
